# Free-Standing Electrospun W-Doped BiVO_4_ Porous Nanotubes for the Efficient Photoelectrochemical Water Oxidation

**DOI:** 10.3389/fchem.2020.00311

**Published:** 2020-04-23

**Authors:** Xiuhua Yuan, Xia Sun, Huawei Zhou, Suyuan Zeng, Bingxin Liu, Xia Li, Dong Liu

**Affiliations:** ^1^School of Mechanical and Automotive Engineering, Liaocheng University, Liaocheng, China; ^2^Department of Chemistry, Liaocheng University, Liaocheng, China; ^3^Key Laboratory of Modern Agricultural Equipment and Technology, Ministry of Education, High Tech Key Laboratory of Agricultural Equipment and Intelligentization of Jiangsu Province, School of Agricultural Equipment Engineering, Jiangsu University, Zhenjiang, China

**Keywords:** BiVO_4_ nanotube, W doping, electrospinning, self-supporting catalyst, photoelectrochemical property

## Abstract

While bismuth vanadate (BiVO_4_) has emerged as a promising photoanode in solar water splitting, it still suffers from poor electron-hole separation and electron transport properties. Therefore, the development of BiVO_4_ nanomaterials that enable performing high charge transfer rate at the interface and lowering charge recombination is urgent needed. Herein, cobalt borate (Co-B) nanoparticle arrays anchored on electrospun W-doped BiVO_4_ porous nanotubes (BiV_0.97_W_0.03_O_4_) was prepared for photoelectrochemical (PEC) water oxidation. One-dimensional, free-standing and porousBiV_0.97_W_0.03_O_4_/Co-B nanotubes was synthesized through electrospun and electrodeposition process. BiV_0.97_W_0.03_O_4_/Co-B arrays exhibit a unique self-supporting core-shell structure with rough porous surface, providing abundant conductive cofactor (W) and electrochemically active sites (Co) exposed to the electrolyte. When applied to PEC water oxidation. BiV_0.97_W_0.03_O_4_/Co-B modified FTO electrode displays high incident photon-to-current conversion efficiency (IPCE) of 33% at 405 nm (at 1.23 V vs. RHE) and its photocurrent density is about 4 times to the pristine nanotube. The higher PEC water oxidation properties of BiV_0.97_W_0.03_O_4_/Co-B porous nanotubes may be attributed to the effectively suppress the electron-hole recombination at electrolyte interface due to its self-supporting core-shell structure, the high electrocatalytic activity of Co and the good electrical conductivity of BiV_0.97_W_0.03_O_4_ arrays. This work offers a simple preparation strategy for the integrated Co-B nanoparticle with BiV_0.97_W_0.03_O_4_ nanotube, demonstrating the synergistic effect of co-catalysts for PEC water oxidation.

## Introduction

Renewable, sustainable and environmental-friendly energy sources (such as solar-hydrogen) are extremely required owing to energy exhaustion and the environmental pollution (Chu and Majumdar, 2012; Dominković et al., 2018). Photoelectrochemical (PEC) water splitting serves as an excellent sustainable and environmentally friendly method to cleanly produce H_2_ from water via solar light (Li et al., 2013; Modestino and Haussener, 2015; Jiang et al., 2017; Lianos, 2017). As the core components in PEC water splitting cell, semiconductor nanomaterials show a decisive influence on the conversion efficiency of solar-to-hydrogen (Alexander et al., 2008; Higashi et al., 2011; Zhang et al., 2013; Li et al., 2014; Shi et al., 2016; Tamirat et al., 2016). Among these candidate semiconductors, the monoclinic BiVO_4_ is most potential for PEC water splitting as it offers moderate band gap (2.4–2.5 eV) and appropriate band-edge positions (Cooper et al., 2014; Tan et al., 2017). Theoretically, the BiVO_4_ can absorb up to 11% of the solar spectrum and produce upwards of 7.5 mA·cm^−2^ of photocurrent (Pihosh et al., 2014). Actually, due to its poor electron mobility (0.044 cm^2^/v·s), small hole collection depth [70 nm (Kim and Lee, 2019)], and excessive surface recombination, bare BiVO_4_ exhibits low efficiency for PEC water splitting, which hampered its use in energy conversion domains (Zachäus et al., 2017). Therefore, the development of BiVO_4_ nanocomposite materials that enable performing high charge transfer rate at the interface and lowering charge recombination is urgent needed for improving the PEC water splitting efficiency.

Currently, the strategy for enlarging charge separation and transport rate mainly involved in the doping foreign elements or co-catalyst (Berglund et al., 2011; Kim et al., 2015; Cheng J. et al., 2016; Zhang et al., 2017). For example, Luo et al. demonstrated an effective BiVO_4_ photoanode through doping with various metal ions using metal-organic decomposition, and found that only doping W^6+^ or Mo^6+^ into V^5+^ site can supply additional free electrons and enhance the PEC photocurrent (Luo et al., 2011; Yang et al., 2017; Xin et al., 2018). Apart from doping foreign elements, the cocatalysts [such as Ni-Bi, Co-Pi, CoMoO_4_, FeOOH and TiO_2_ (Zhou et al., 2012; Cheng B. Y. et al., 2016; Wang et al., 2017; Du et al., 2018)] can improve the water oxidation kinetics by reducing the activation energy of the rate-determining step of the four electron oxidation process. Significantly, PEC water splitting efficiency mainly depended both on charge transfer and on water oxidation kinetic. In this regard, the integration of doping foreign elements and cocatalyst on the BiVO_4_ photoanode are more required for the higher PEC water splitting efficiency.

Apart from above factors, nanostructuring is an effective approach to reduce bulk recombination by shortening the diffusion length for charge carriers (Zhang et al., 2014a,b; Han et al., 2017). The BiVO_4_ with one-dimensional (1D) morphologies (such as nanowire, nanotube and nanofiber), which has long axial-ratio and high active sites, is a promising photocatalyst (Boettcher et al., 2010; Hernández et al., 2017). These nanostructures, especially nanotube, can provide a large specific surface area, and also short the carrier diffusion length. Recent study demonstrated that 1D nanomaterial has been proved to be outstanding for solar-to-hydrogen conversion (Yao et al., 2019). Significantly, electrospun is a simple, flexible and efficient technology to deal with polymer/inorganic materials into three-dimensional nanofibers with controllable composition, diameter and porosity, and has been concerned in the photovoltaics, chemical sensors, and photocatalysis owing to the one-dimensional open structure, large surface areas, and high porosity (Kumar et al., 2014). While electrospun had been used to fabricate BiVO_4_ nanofibers for photocatalysis (Yoon et al., 2015), no studies have been reported on fabricating BiVO_4_ nanotubes as a photoanode for water splitting, and the effect of doping and cocatalyst on the nanotubes.

Here, one-dimensional, free-standing and porous BiV_0.97_W_0.03_O_4_/Co-B nanotubes were synthesized through electrospun and electrodeposition process. BiV_0.97_W_0.03_O_4_/Co-B arrays exhibited a unique self-supporting core-shell structure with rough porous surface, providing abundant conductive cofactor (W) and electrochemically active sites (Co) exposed to the electrolyte. When applied to PEC water oxidation, BiV_0.97_W_0.03_O_4_/Co-B modified FTO electrode displayed higher incident photon-to-current conversion efficiency (IPCE) of 33% at 405 nm (1.23 V vs. RHE), and its photocurrent density is about 4 times to the pristine nanotube. The higher PEC water oxidation properties of BiV_0.97_W_0.03_O_4_/Co-B porous nanotubes may be attributed to the effectively suppress the electron-hole recombination at electrolyte interface due to its self-supporting core-shell structure, the high electrocatalytic activity of Co and the good electrical conductivity of BiV_0.97_W_0.03_O_4_ arrays. This work offers a simple preparation strategy for the integrated Co-B nanoparticle with BiV_0.97_W_0.03_O_4_ nanotube, demonstrating the synergistic effect of co-catalysts for PEC water oxidation.

## Materials and Methods

**Figure 1:**
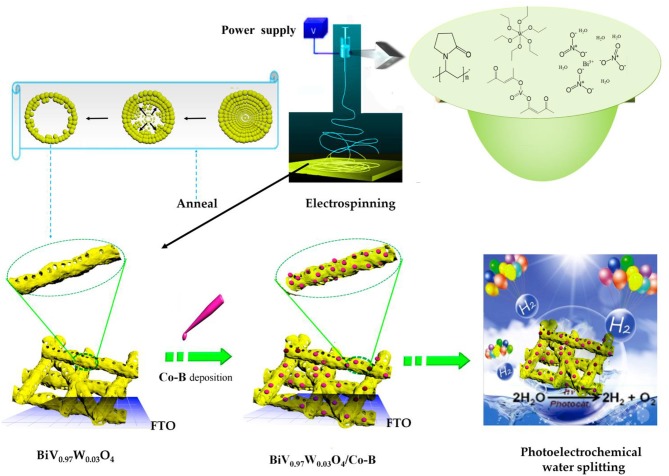
Schematic illustration of BiV_0.97_W_0.03_O_4_/Co-B nanotube fabrication process.

The BiV_0.97_W_0.03_O_4_/Co-B nanotube was fabricated through a strategy including three sequential steps: electrospun, high-temperature annealing, and electrodeposition, as illustrated in Figure 1.

### Reagents

The bismuth nitrate (Bi(NO_3_)_3_·5H_2_O, Aladdin, China), vanadium(IV)-oxy acetylacetonate (VO(acac)_2_, J&K Chemical Ltd, China), tungsten ethoxide (W(OC_2_H_5_)_6_, Alfa-aesar, USA), cobaltous nitrate(Co(NO_3_)_2_·6H_2_O, Aladdin, China), polyvinylpyrrolidone (PVP, K-90, Mw = 1,300,000, J&K Chemical Ltd, China), N, N-Dimethylformamide (DMF), and acetylacetone (CH_3_COCH_2_COCH_3_) were chemical reagents. All the chemical reagents were of analytical grade, and water used in all experiment was distilled and deionized.

### Preparation of BiV_0.97_W_0.03_O_4_/Co-B Porous Nanotube

Firstly, Bi(NO_3_)_3_∙5H_2_O and VO(acac)_2_ were added and dissolved into a mixture of acetylacetone and DMF. Subsequently, according to the stoichiometric ratio of Bi: V: W = 100: 97: 3, the W(OC_2_H_5_)_6_ was added and stirred for 1 h to form metal ion complex (Figure S1A). Then, the PVP was added and stirred 12 h, forming the homogeneous and stable precursor solution for electrospinning.

The electrospinning experiment was performed on a self-made instrument, which consisted of a syringe, a grounded collector, and a high-voltage supply. The homogeneous solution was transformed into the plastic syringe equipped with a needle (inner-diameter 0.4 mm) as the spinneret. The counter plate as collector was covered with aluminum foil, where fluorine doped tinoxide glass (FTO, 3 × 2.5 cm^2^, OPV-FTO-22-07) was also arranged to collect the nanofiber. The electrospinning was carried out at tip-collector spacing of 15 cm, accelerating voltage of 19 kV, flow rate of 0.4 mL/h, and relative humidity of 30%. After electrospinning for 30 min, the nanofiber mats collected on FTO were dried at 110°C for 10 h, which was shown in Figure S1B. Based on the TG results (see Figure S2), the thermal annealing was carried out at 490°C for 1.5 h. After naturally cooling down to room temperature, the electrodes of BiV_0.97_W_0.03_O_4_ nanotube was successfully obtained, which was shown in Figure S1C. For comparison, some electrodes of pristine BiVO_4_ nanotubes were also prepared.

The Co-B was loaded on BiV_0.97_W_0.03_O_4_ nanotube by electrodeposition. A three-electrode system was employed with an as-prepared nanotube electrode (working electrode), a Ag/AgCl reference electrode, and a platinum counter electrode. The electrolyte was 0.5 mM Co(NO_3_)_2_·6H_2_O in 0.1 M potassium borate buffer (pH = 8.50). The electrodeposition was carried out using an electrochemical workstation (Zahner Zennium) at 0.70 V vs. Ag/AgCl for 1 min.

### Characterization

The morphology of nanotube was investigated by scanning electron microscopy (SEM, Zeiss Merlin), energy-dispersive X-ray spectroscopy (EDS), and transmission electron microscope (TEM, JEM-2100). The structural properties were characterized by X-ray photoelectron spectroscopy (XPS, ESCALAB Xi^+^) and X-ray diffraction (XRD, Bruker Smart-1000CCD diffractometer with Cu Kα radiation, λ = 1.5406 nm). The thickness of photoanode was measured by step profiler (KLA-Tencor D100). Light absorption was measured using a UV–visible spectrophotometer (PE lambda 750) by measuring the reflectance with an integrated sphere attachment. The PEC experiments of as-prepared BiVO_4_ and BiV_0.97_W_0.03_O_4_ nanotubes were performed in 0.5 M Na_2_SO_4_ electrolyte(PH = 7.00) on a electrochemical workstation (Zahner Zennium, Germany) in a three-electrode PEC cell. While the PEC performance of BiV_0.97_W_0.03_O_4_/Co-B nanotube was measured in the same PEC cell with 0.1 M borate electrolyte (pH = 8.50). The PEC cell was composed of Ag/AgCl reference electrode, a platinum counter electrode and working electrode (the as-prepared nanotubes on FTO), respectively. The white light LED (average λ = 536 nm, P = 100 mW·cm^−2^, Figure S3) and 300 W Xe lamp (CEL-HXF300-T3, P = 100 mW·cm^−2^, λ > 420 nm) were used as illumination source. For incident photon-to-current efficiency (IPCE) measurements, a Zahner tunable light source system, model CIMPS TLS03, was employed to exhibit a LED array for monochromatic light excitation. The chopped light voltammetry measurement was carried out with scan speed of 10 mV/s and light period time of 8 s. The photoelectrochemical impedance spectroscopy (PEIS) was performed from 1 to 10^5^ Hz frequency with 5 mV amplitude. The Mott-Schottky (M-S) analysis under dark condition was carried out at frequencies 1 kHz with a step width of 50 mV/s.

## Results and Discussions

### Morphology and Structure of Nanotube

**Figure 2:**
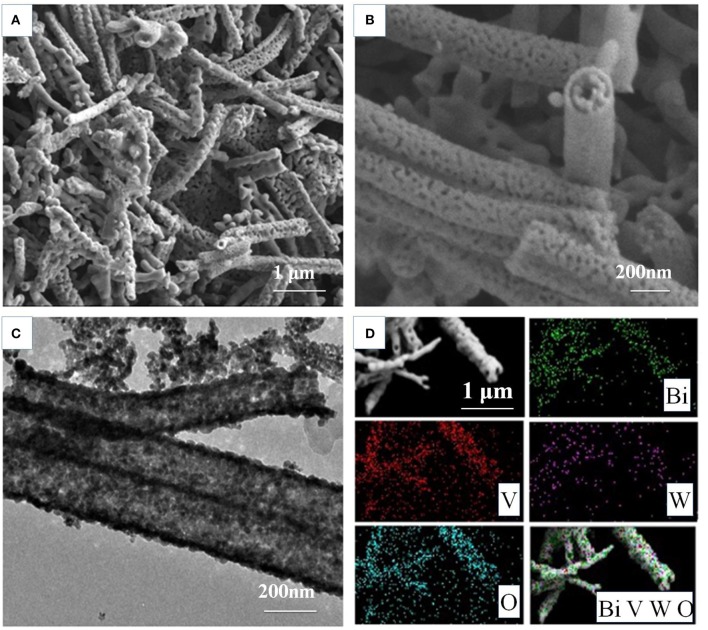
Morphology characterizationofthe BiV_0.97_W_0.03_O_4_ nanotubes: **(A,B)** SEM images; **(C)** the corresponding TEM images; **(D)** the elemental mapping Bi, V, O, and W, respectively.

The morphology of the as-prepared BiV_0.97_W_0.03_O_4_ nanotube are inspected by SEM and TEM measurements. Figure S1C shows the photograph of BiV_0.97_W_0.03_O_4_ film on FTO glass by elelctrospinning, which shows the good light transmission. Figure 2A shows that the non-woven film is formed by randomly oriented nanotubes, and the nanotubes possess porous hollow tubular structure which has sufficiently large surface active sites for electron and hole separations. The high magnification image of Figure 2B reveals that the porous nanotube exhibits a diameter ranging from 150 to 300 nm, and further verifies a porous structure. Figures 2B,C reveal the wall thickness of nanotubes are about 15 and 40 nm, respectively, which indicate a non-uniform shell structure throughout the nanotube. The elemental mapping is shown in Figure 2D for Bi, V, O, and W, respectively, which further verifies the homogeneous distribution of elements within the nanotube.

**Figure 3:**
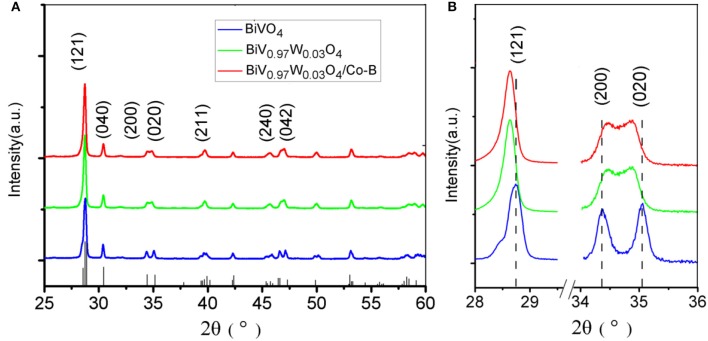
**(A)** XRD patterns of different photoanodes, **(B)** the highlight peaks that changing with W doping.

**Figure 4:**
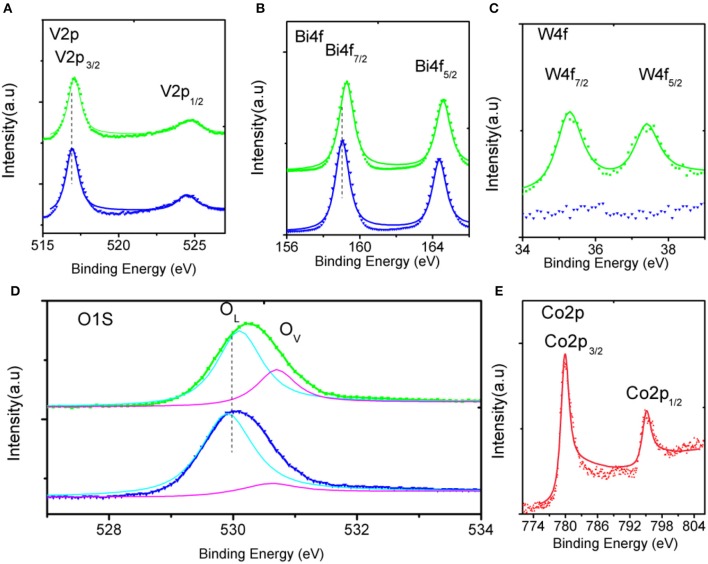
High-resolution XPS spectra of **(A–D)** in the pristine (blue), doped (green) BiVO_4_ nanotubes, and **(E)** in Co-B.

The crystal structures of the as-fabricated electrodes are further characterized by XRD (Figure 3). In the pristine BiVO_4_ nanotube pattern, the four diffraction peaks in 28.5, 30.5, 34.5, and 35.2° are related to the crystal planes (121), (040), (200), and (020) of the monoclinic BiVO_4_, respectively (JCPDS 014-0688). After doping W, the predominant (121) and (020) peaks move toward the negative direction, and the (200), (020) peaks closely shift toward each other. This result can be caused by partial V^5+^sites being occupied by W^6+^, because of Bi (1.03 Å) >W (0.42 Å) > V (0.355 Å), making W^6+^ -doping feasible. The peaks merging of (200) and (020) can be ascribed to a slight lattice change from monoclinic to tetragonal symmetry. No Co-B diffraction peaks can be found in the XRD patterns because of amorphous Co-B (Kanan and Nocera, 2008). The chemical composition is also verified by XPS, which are presented in Figure 4. In the pristine BiVO_4_ nanotube, the two main asymmetric peaks of Bi4f_7/2_ and Bi4f_5/2_ can be found at 159.0 and 164.7 eV, respectively, corresponding to the Bi^3+^ oxidation state; Meanwhile, the two asymmetric peaks of V2p_3/2_ and V2p_1/2_ can be found at 517.0 and 524.9 eV, respectively, which is corresponding to a V^5+^ oxidation state. In the BiV_0.97_W_0.03_O_4_ nanotube, the W4f_7/2_ and W4f_5/2_ peaks located at 35.2 and 37.8 eV, respectively, suggesting the oxidized state of W (W^6+^) to substitute V^5+^ atoms on the surface of the BiVO_4_ photoanode, and the positive charge will be compensated by free electrons. The asymmetrical O1s peak (Figure 4D) in the range of 528–534 eV is fitted into two components centered at 529.9, 530.7 eV which are assigned to the lattice species (O_L_) and oxygen vacancies (O_V_), respectively. It is worth noting that BiV_0.97_W_0.03_O_4_ nanotube possesses more oxygen vacancies than the pristine sample. Moreover, the binding energies of the Bi4f, V2p and O1s slightly shift toward positive direction compared to the pure BiVO_4_, due to the higher electronegativity of the W-dopant (Zhang et al., 2017). As shown in Figure 4E, the two main asymmetric peaks of Co2p_3/2_ and Co2p_1/2_ can be found at 780.3 and 795.0 eV, respectively, both corresponding to the Co^2+^. Together, the XRD and XPS results confirm that the W^6+^ cation is expectantly doped into the BiVO_4_ lattice and also slightly deform the monoclinic structure.

### PEC Performance

**Figure 5:**
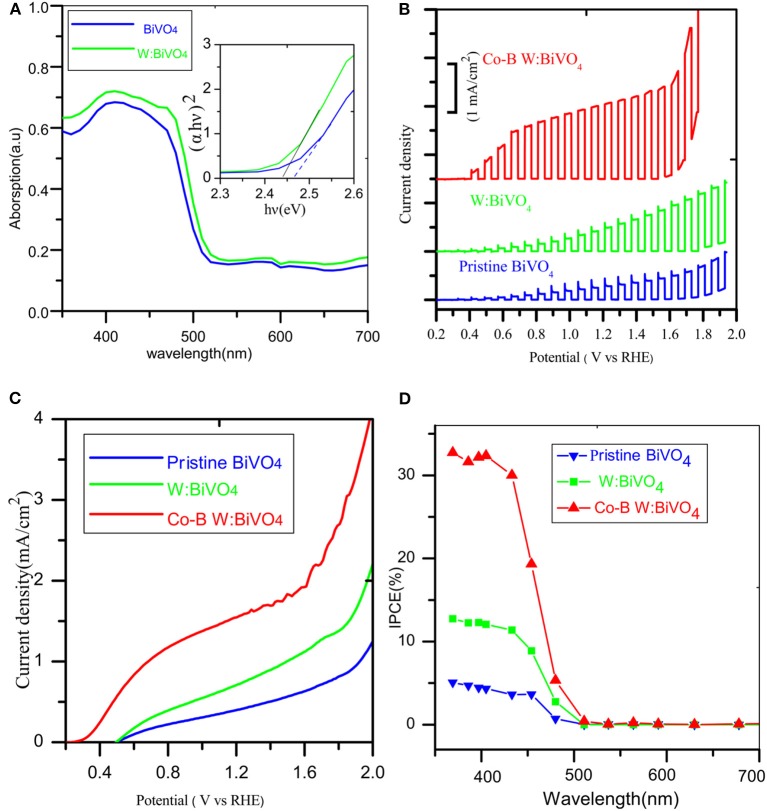
**(A)** UV–Vis diffuse reflection spectra of the photoanodes, **(B)** Chopped photocurrent vs. potential curves under white light LED, **(C)** photocurrent vs. potential (J-V) curves under AM 1.5 G illumination **(D)** IPCE spectrum.

**Figure 6:**
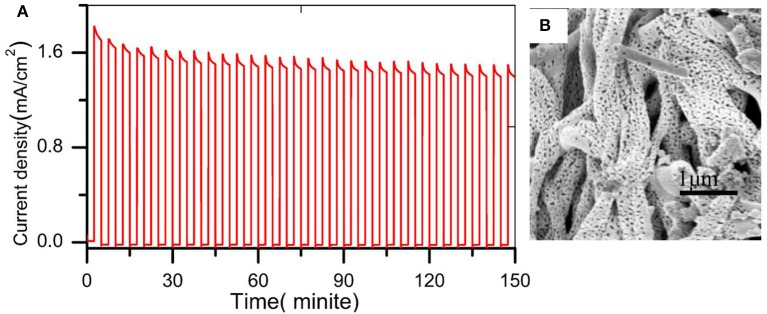
**(A)** photocurrent vs. time curve under white light LED chopping illumination at 1.23 V vs. RHE **(B)** SEM image after PEC water oxidation.

The band gap was estimated using UV-visible spectroscopy in diffuse reflectance mode. As shown in Figure 5A, there was no obvious enhancement of the UV absorption intensity after doping W element, indicating that the W element was not essential for light absorption. The absorption edges were located at about 505 nm (2.45 eV) and its electrode modification layer thickness was about 800 nm. To study the PEC performance of BiVO_4_ nanotube, the linear sweep voltammetry under light illumination was carried out. Figure 5B depicts the current density under intermittent light irradiation of pristine BiVO_4_, BiV_0.97_W_0.03_O_4_, and BiV_0.97_W_0.03_O_4_/Co-B nanotubes. The photocurrents promptly increase when the light is on, and drop when the light is off over a wide potential range, which imply that the photocurrent is generated under light irradiation. It can be also seen that the transient photocurrent peak upon turning the light off/on, which indicates the accumulation of holes at the space charge layer under prolonged irradiation, and the back recombination of electrons with the accumulated holes. Figure 5C shows the current-potential (J-V) curves under AM 1.5G irradiation. The photocurrents of BiVO_4_, and BiV_0.97_W_0.03_O_4_ nanotube increase slowly with the increase of applied bias with an onset potential of 0.48 V vs. RHE, and yield photocurrent density of 0.39 and 0.65 mA/cm^2^ at 1.23 V vs. RHE, respectively. After depositing Co-B on BiV_0.97_W_0.03_O_4_ nanotube, the photocurrent increase quickly with the increase of applied bias with an onset potential of 0.35 V vs. RHE, and yields a photocurrent density of 1.59 mA/cm^2^ at 1.23 V vs. RHE. Notably, the photocurrent density of BiV_0.97_W_0.03_O_4_/Co-B nanotube represented enhancement of about 400% to that of pristine nanotube. Then, the IPCE using the Zahner tunable light source system at 1.23 V vs. RHE was carried out. As shown in Figure 5D, The IPCE values decrease with increasing wavelength and are zero at 510 nm (2.43 eV) for these nanotubes, which suggests no difference in their band gap energy. Significantly, the BiV_0.97_W_0.03_O_4_/Co-B nanotube exhibits the highest IPCE values reaching up to 33% at 405 nm, as compared to those of pristine nanotubes (4.8% at 405 nm) and BiV_0.97_W_0.03_O_4_ nanotubes (13% at 405 nm). It demonstrates that the IPCE value of BiV_0.97_W_0.03_O_4_/Co-B nanotube is enhanced about 6 times to that of pristine nanotube, owing to the enhanced charge transport and separation. Figure 6A shows the photocurrent–time curve of BiV_0.97_W_0.03_O_4_/Co-B nanotube under chopped illumination at 1.23 V vs. RHE. The photocurrent density of BiV_0.97_W_0.03_O_4_/Co-B nanotube was decreased by 13% until 150 min, which indicated its high stability. After PEC test (about 2.5 h), it remains the same surface morphology of porous nanotube (Figure 6B) as before. According to the value of photocurrent density and IPCE, the BiV_0.97_W_0.03_O_4_/Co-B nanotube exhibits the highest PEC performance ever achieved for BiVO_4_ nanofiber using electrospinning (Yoon et al., 2015; Antony et al., 2016; Cheng J. et al., 2016; Liu et al., 2017; He et al., 2018; Li et al., 2019). Table S1 shows the comparison of photocurrent data reported in the literature with the photocurrent value obtained in the present study. The nanotube-based BiVO_4_ photoanode exhibit lower photocurrent density than state-of-the-art photoanode by electrodeposition method about 1.05 mA·cm^−2^ at 1.23 V vs. RHE. Therefore, an optimization of the film thickness in order to increase amount of active material per area unit is highly desirable, and will be shown and discussed later on.

**Figure 7:**
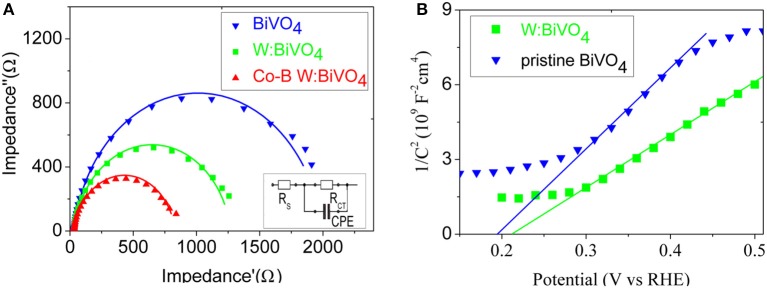
**(A)** Nyquist plots at 1.23 V vs. RHE under white light LED, and the inset display an equivalent circuit model; **(B)** Mott-Schottky plots under dark condition.

In order to identify the influence of W^6+^ and Co-B on the charge transport kinetics of the BiVO_4_ nanotube, the analysis of PEIS is carried out at 1.23 V vs. RHE. Figure 7A shows the Nyquist plots of the pristine BiVO_4_, BiV_0.97_W_0.03_O_4_, and BiV_0.97_W_0.03_O_4_/Co-B nanotubes, respectively, and the inset of Figure 7A shows the equivalent electrical circuit. In this equivalent electrical circuit, theR_CT_ denotes the charge transfer resistance from the BiVO_4_ photoanode to electrolyte solution, the CPE represents the constant phase element for the electrolyte/photoanode interface, and the Rs is the resistance associated with FTO substrates, the electrolyte, and wire connections in the whole circuit. As shown in Table S2, the total error was below 3%, which presents the Nyquist plots can be fitted well with the equivalent circuit model. The R_S_ values of the pristine BiVO_4_, BiV_0.97_W_0.03_O_4_, and BiV_0.97_W_0.03_O_4_/Co-B electrodes are about 14.5 Ω, which indicates the good conductivity of the FTO substrate. The R_CT_ values of the pristine BiVO_4_, BiV_0.97_W_0.03_O_4_, and BiV_0.97_W_0.03_O_4_/Co-B are 1964, 1442, and 610 Ω, respectively. The BiV_0.97_W_0.03_O_4_ electrode has a smaller R_CT_ value than the pristine electrode, which implies the electron transfer in the nanotubes improved by W doping. The BiV_0.97_W_0.03_O_4_/Co-B nanotube shows the smallest R_CT_ value, which indicates that the surface recombination is suppressed by the Co-B nanoparticle. These results well explain the enhancement of photocurrent density after doping W^6+^and depositing Co-B. To further evaluate the effect of W doping on the electronic properties of BiVO_4_ nanotube, the M-S measurements were carried out (Figure 7B). The flat band potential (E_fb_) can be calculated using equation (see supporting information in ESI^†^). The E_fb_ of pristine and W-doped BiVO_4_ nanotubes are 0.215, 0.196 V vs. RHE, respectively. Moreover, their carrier densities are 6.4 × 10^19^, 8.9 × 10^19^ cm^−3^, respectively. This result provides direct evidence that W element is effectively doped into BiVO_4_ lattice, and can effectively elevate the donor density, which result in the efficient electron transportation.

**Figure 8:**
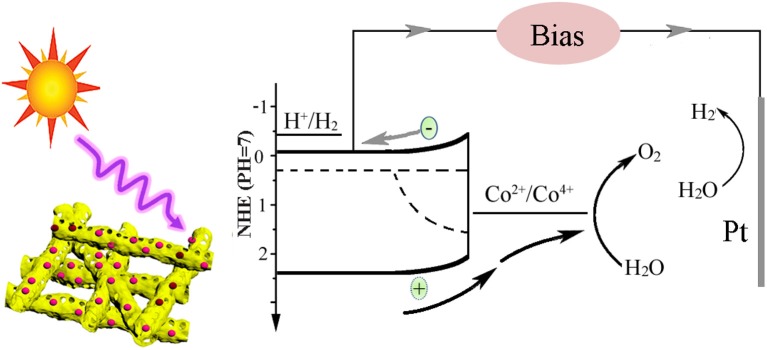
Schematic illustration of the transport process of photo-generated carriers.

Based on the above results, the charge transfer process in the BiV_0.97_W_0.03_O_4_/Co-B nanotube photoanode can be demonstrated in Figure 8. Under light illumination, the BiV_0.97_W_0.03_O_4_ nanotube can absorb the photons, and generate electron–hole pairs. Then the photogenerated electrons and holes migrate to the surface of photoanode, and inject into the electrolyte to generate hydrogen and oxygen, respectively. From Figure S4, we can see that there are a lot of recombination of photogenerated electron–hole pairs both in bulk and surface during the overall reaction. The results of PEC test show that the BiV_0.97_W_0.03_O_4_/Co-B nanotube demonstrated better photocatalytic activity compared with other nanofibers that have ever been reported (Yoon et al., 2015; Antony et al., 2016; Cheng J. et al., 2016; Liu et al., 2017; He et al., 2018; Li et al., 2019). The reasons can be explained as follows: first of all, the nanotube with large interface area and the porous structure can keep in good contact with the electrolyte, and enrich the active sites. Meanwhile, the W^6+^-doping at V^5+^ sites in the BiV_0.97_W_0.03_O_4_ nanotube can increase the donor density for the host lattice, and reduce the effective mass of electron, resulting in a higher probability of reaching the active sites of electrode /electrolyte; the nanotube can also minimize the distance of electrons diffusing to the FTO substrate, thereby decreasing the bulk electron/hole recombination; the Co-B cocatalyst can also efficiently extracts photo generated holes from the BiV_0.97_W_0.03_O_4_ nanotube, and store long-lived holes as Co^4+^ species. Thus, the more holes migrate to the electrode/ electrolyte interface, and the back electron/hole recombination in the space charge layer is significantly retarded. In summary, porous nanotube, doping, and cocatalyst should account mainly for the enhanced PEC performance of BiV_0.97_W_0.03_O_4_/Co-B nanotube photoanode.

## Conclusions

In summary, the cobalt borate (Co-B) nanoparticle arrays anchored on W-doped BiVO_4_ porous nanotubes (BiV_0.97_W_0.03_O_4_) with large specific surface area and short diffusion length have been successfully synthesized by electrospun and electrodeposition process. The as-prepared BiV0_.97_W_0.03_O_4_/Co-B arrays exhibit a unique self-supporting core-shell structure with rough porous surface, providing abundant active sites exposed to the electrolyte. Their diameters range from 150 to 300 nm, and wall thicknesses range from 10 to 40 nm. XPS and XRD reveal that the W-cations are doped into lattice and also slightly deform the monoclinic structure. PEIS and M-S measurements reveal that doping a small amount (3 atom%) of W^6+^ can effectively increase carrier density and reduce the charge transfer resistance. The photoanode of BiV_0.97_W_0.03_O_4_/Co-B nanotube possesses the IPCE of 33% at 405 nm at 1.23 V vs. RHE, and its photocurrent density is about 4 times to that of the pristine nanotube. This enhancement is attributed to the suppression of surface electron/hole recombination, and the higher bulk electron transport. These results offer a simple preparation strategy for the integrated Co-B nanoparticle with BiV_0.97_W_0.03_O_4_ nanotube, demonstrating the synergistic effect of co-catalysts for PEC water oxidation.

## Data Availability Statement

All datasets generated for this study are included in the article/Supplementary Material.

## Author Contributions

XY planned the experimental work, wrote the manuscript, and helped in the analysis. XS, HZ, and SZ helped in the analysis, and explained the results. XL and BL writing of original draft manuscript. DL helped in the experimental work and explained the results.

## Conflict of Interest

The authors declare that the research was conducted in the absence of any commercial or financial relationships that could be construed as a potential conflict of interest.
