# Supplementary material for: Free-Standing Electrospun W-Doped BiVO4 Porous Nanotubes for the Efficient Photoelectrochemical Water Oxidation
Source: Front Chem. 2020 Apr 23;8:311. doi: 10.3389/fchem.2020.00311 (PMC7192020; doi:10.3389/fchem.2020.00311)
Supplement: Supplementary file 1 [file Table_1.docx]

Supplementary Material

**Table of Contents**

**FIGURE S1** Digital Micrograph of (a) solution precursor, (b) PVP- BiV_0.97_W_0.03_O_4_ nanofiber mats before annealing and (c) BiV_0.97_W_0.03_O_4_ nanotube mats after annealing...........................**2**

**FIGURE S2** Thermogravimetric analysis of the crystallization of BiV_0.97_W_0.03_O_4_ nanotube...............**3**

**FIGURE S3** Emission spectrum of the white light LED from Zahner -Elektrik...................................**4**

**FIGURE S4** Linear sweep voltammetry curves with (A) and without (B) the addition of Na_2_SO_3_ under white light LED………………………………………………………………………. 5

**Table S1.** Comparison of photocurrent data reported in the literature with the photocurrent value obtained in the present study ………………………………………………………………...6

**Table S2.** Output of the equivalent circuit model from the nyquist plot................................................**7**

**Calculations formula.**...........................................................................................................................**8**


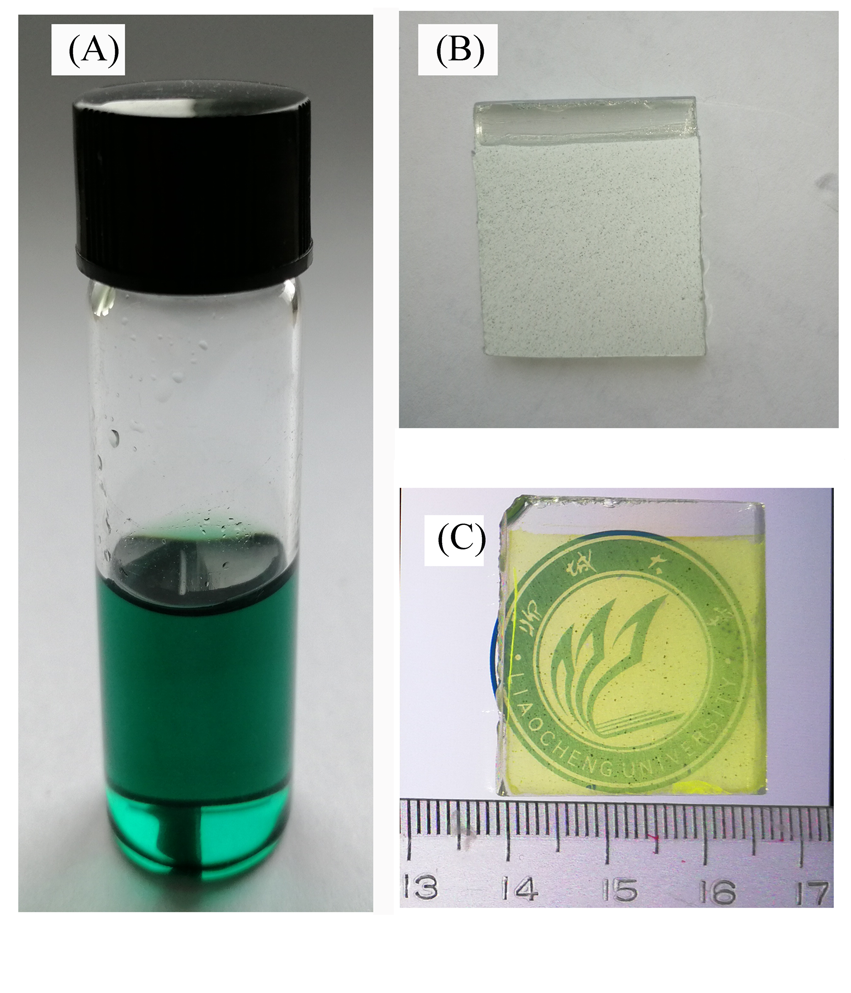


**FIGURE S1** Digital Micrograph of (A) solution precursor, (B) PVP-BiV_0.97_W_0.03_O_4_ nanofiber mat before annealing and (C) BiV_0.97_W_0.03_O_4_ nanotube mat after annealing


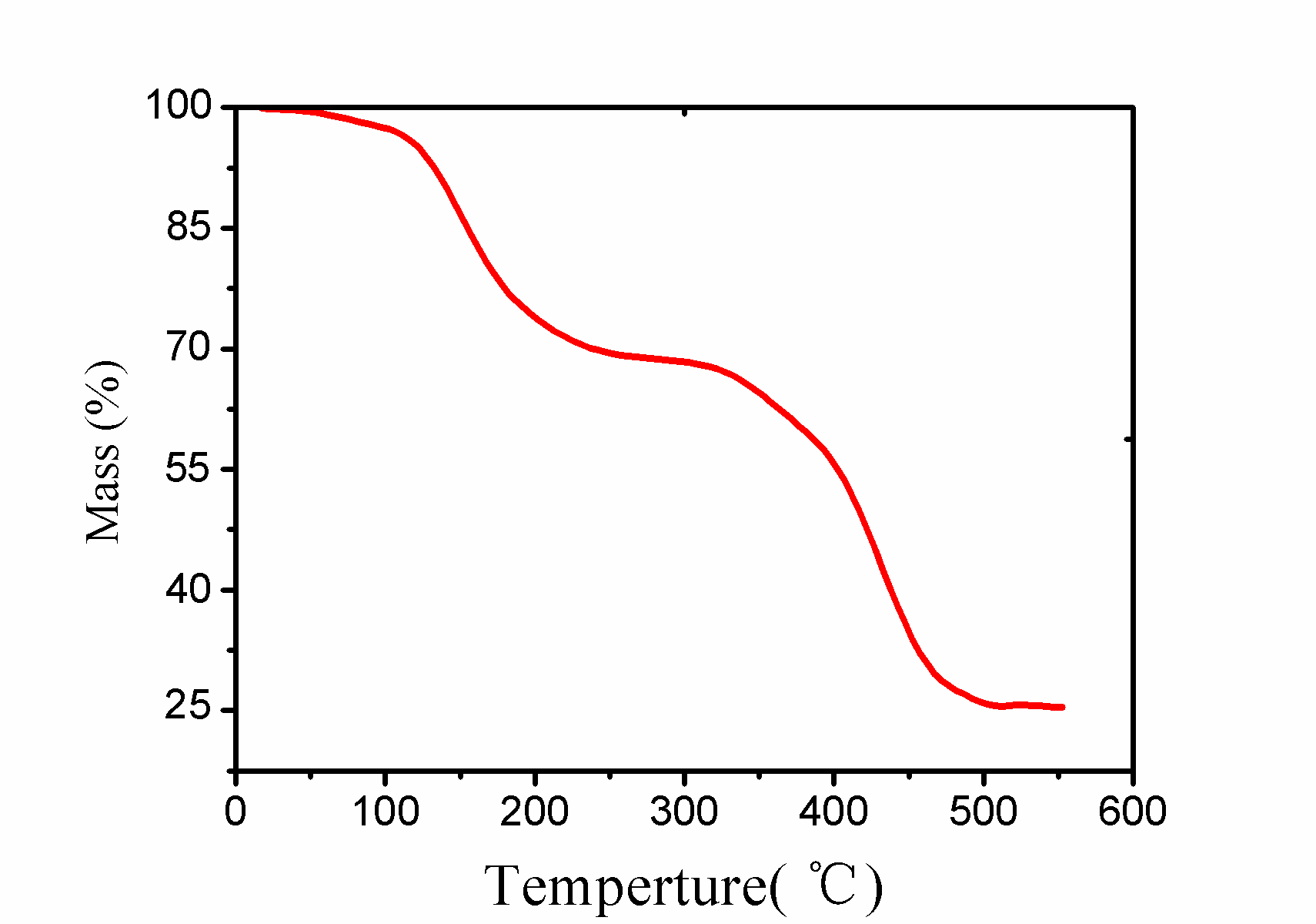


**FIGURE S2.** Thermogravimetric analysis of the crystallization of BiV_0.97_W_0.03_O_4_ nanotubes


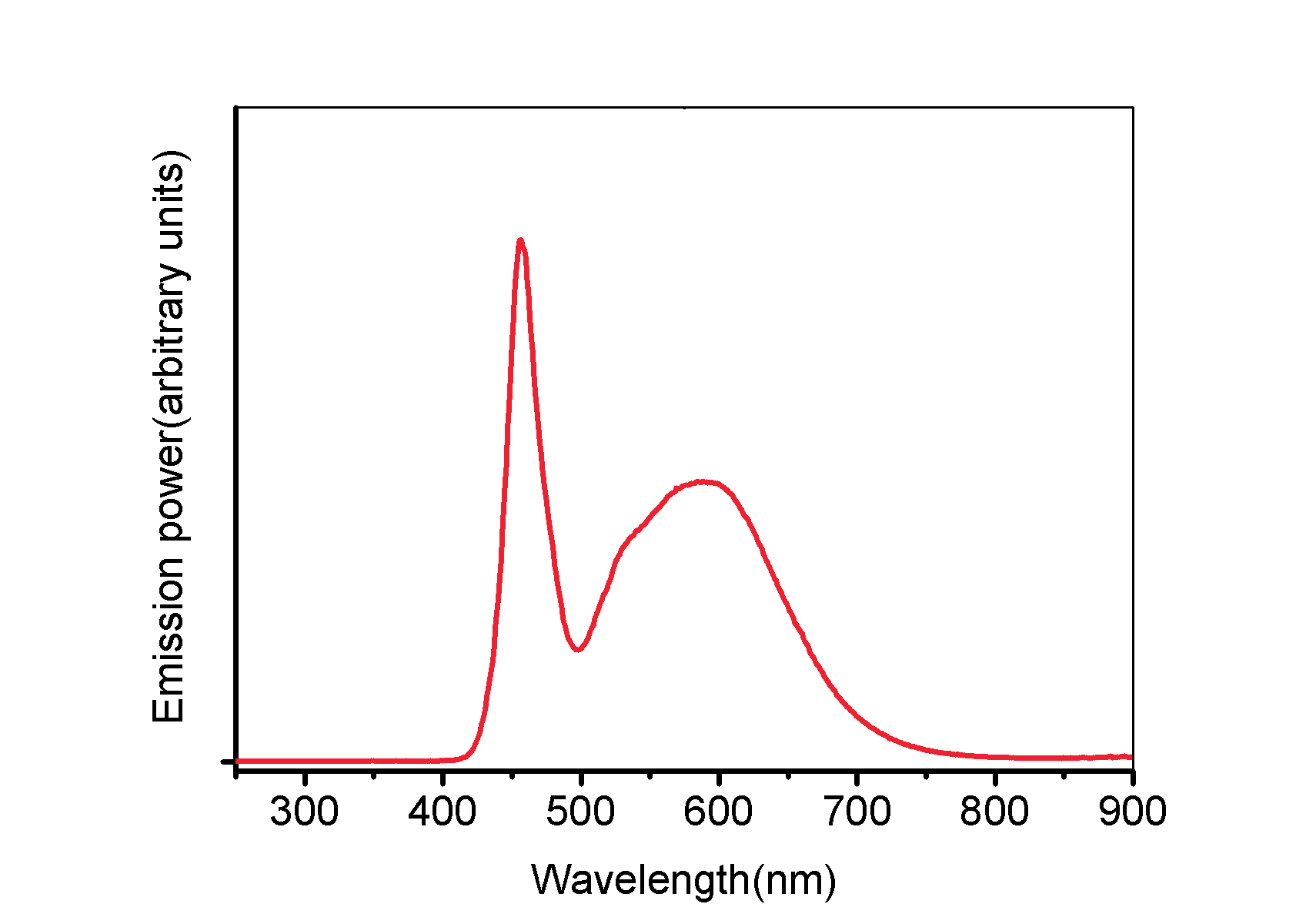


**FIGURE S3** Emission spectrum of the white light LED from Zahner-Electric


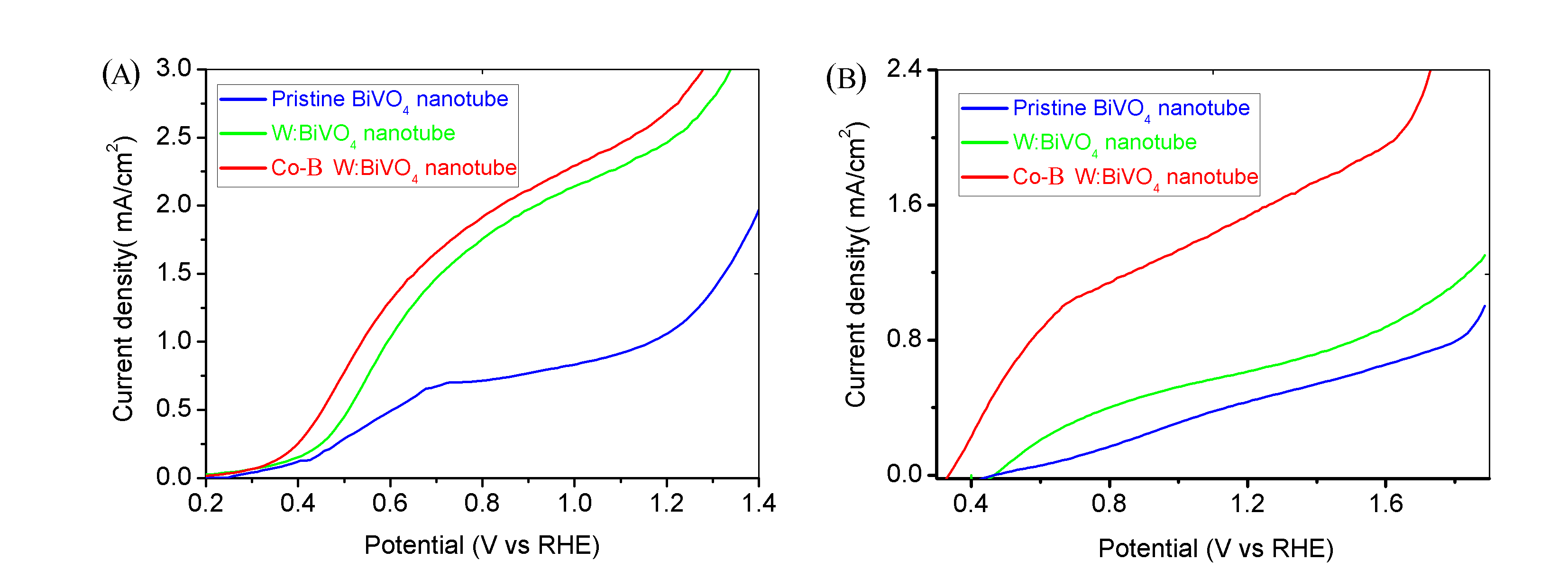


**FIGURE S4** Linear sweep voltammetry curves with (A) and without (B) the addition of Na_2_SO_3_ under white light LED

**Table S1.** Comparison of photocurrent data reported in the literature with the photocurrent value obtained in the present study

| Fabrication method | BiVO_4_ Photoanode | Thickness  (μm) | Band gap  (eV) | Performance  (at 1.23V RHE) | Ref |
| --- | --- | --- | --- | --- | --- |
| Hydrothermal reaction | pseudo-flower shaped | / | 2.41 | 0.06 mA/cm^2^ | 1 |
| Pulsed laser deposition | Pristine nanofilm | 0.2 | about 2.38 | 0.27 mA/cm^2^ | 2 |
| Metal organic decomposition | Pristine nanofilm | 0.18 | 2.49 | 0.30 mA/cm^2^ | 3 |
| Single-source precursors drop-casting | Pristine nanofilm | 1.44 | about 2.43 | 0.4 mA/cm^2^ | 4 |
| **Electrospun** | **Pristine nanotube** | **0.8** | **2.46** | **0.39 mA/cm^2^** | **This work** |
| Electrodeposition + etch | Pristine porous film | 1.25 | about 2.43 | 1.05 mA/cm^2^ | 5 |
| PS templated sol-gel | decorated with Al-doped ZnO networks | 0.9 | about 2.33 | 1.50 mA/cm^2^ | 6 |
| **Electrospun** | **BiV_0.97_W_0.03_O_4_/Co-B nanotube** | **0.8** | **2.44** | **1.59 mA/cm^2^** | **This work** |

**Table S2.** Output of the equivalent circuit model from the nyquist plot

| **R/**Ω | Pristine BiVO_4_ | BiV_0.97_W_0.03_O_4_ | BiV_0.97_W_0.03_O_4_/ Co-B |
| --- | --- | --- | --- |
| **R_S_** | 15.3 | 14.1 | 14.2 |
| **R_CT_** | 1964 | 1442 | 610 |
| **Overall Error** | 2.99% | 2.65% | 2.19% |

All measurements were carried out with Ag/AgCl reference electrode, but results in this work are presented against reversible hydrogen electrode (RHE) by using the equation:

*E_RHE_* = *E_Ag/Agcl_*+0.196+0.059·PH (1)

*E_RHE_* = *E_NHE_* +0.059·PH (2)

The IPCE values are calculated by using the equation:

IPCE=(1240*I*)/(*λ P*) (3)

Where *I* is the photocurrent density (mA/cm^2^), *λ* is the incident light wavelength (nm), and *P* is the power density of monochromatic light at a specific wavelength.

The flat band potential (E_fb_) can be estimated from the intercept of the MS plot by using the following equation:

$\frac{1}{C^{2}}$ = $\frac{1}{q\varepsilon\varepsilon_{0}N_{d}}\left( E-E_{fb}-\frac{KT}{q} \right)$ (4)

where *C* is the specific capacitance (*F/cm^2^*), *q* is the electron charge, *ε* is the dielectric constant of the material, *ε_0_* is the permittivity of the vacuum, *N_d_* is the carrier density, E is the applied potential, *k* is the Boltzmann’s constant, and T is the temperature (K).

Reference

[1] Khan, I., Ali, S., Mansha, M., and Qurashi, A. (2017). Sonochemical assisted hydrothermal synthesis of pseudo-flower shaped bismuth vanadate (BiVO_4_) and their solar-driven water splitting application. Ultrasonics Sonochemistry, 36, 386-392. doi: 10.1016/j.ultsonch. 2016. 12. 014

[2] Song, J., Cha, J., Lee, M. G., Jeong, H. W., Seo, S., and Yoo, J. A. (2017). Template-engineered epitaxial BiVO_4_ photoanodes for efficient solar water splitting. J. Mater. Chem. A. 5, 18831-18838. doi: 10.1039/C7TA04695b.

[3] Byun, S., Kim, B., Jeon, S., and Shin, B. (2017). Effects of a SnO_2_ hole blocking layer in a BiVO_4_ -based photoanode on photoelectrocatalytic water oxidation. J. Mater. Chem. A, 5(15), 6905-6913. doi:10.1039/C7TA00806F.

[4] Lu, H., Andrei, V., Jenkinson, K.J., and Regoutz, A., et al (2018). Single‐Source bismuth (transition metal) polyoxovanadate precursors for the scalable synthesis of doped BiVO_4_ photoanodes. Adv. Mater., 30(46), 1-6. doi:10.1002/adma.201804033

[5] Mcdonald, K.J., and Choi, K.S. (2012). A new electrochemical synthesis route for a BiOI electrode and its conversion to a highly efficient porous BiVO_4_ photoanode for solar water oxidation. Energ. Environ.Sci., 5(9), 8553-8557.doi: 10.1039/c2ee22608a.

[6] Zhang, L., Reisner, E., and Baumberg, J.J. (2014). Al-doped ZnO inverse opal networks as efficient electron collectors in BiVO_4_ photoanodes for solar water oxidation [J]. Energ. Environ. Sci., 7(4), 1402-1408. doi: 10.1039/c3ee44031a.
